# Quantitative ^19^F MRI and CT tracking of the microencapsulated stem cells in a rabbit peripheral arterial disease model

**DOI:** 10.1186/1532-429X-16-S1-P61

**Published:** 2014-01-16

**Authors:** Guan Wang, Yingli Fu, Steven Shea, Judy Cook, Dara Kraitchman

**Affiliations:** 1 Radiology, Johns Hopkins University, Baltimore, Maryland, USA; 2 Electrical and Computer Engineering, Johns Hopkins University, Baltimore, Maryland, USA; 3 Molecular and Comparative Pathobiology, Johns Hopkins Univerisity, Baltimore, Maryland, USA; 4 Corporate Technology, Siemens Corporation, Baltimore, Maryland, USA

## Background

Nearly 12% of Americans suffer from peripheral arterial disease (PAD) and many are not eligible for conventional treatment. Transplanting stem cells (SC) in microcapsules impregnated with X-ray/MR-visible contrast agents (XMRCaps) offers a novel means for PAD therapy to avoid immunorejection and enable tracking using non-invasive imaging modalities. Here we explore quantitative serial cell tracking of XMRCaps using conventional c-arm CT and ^19^F-MRI containing either human or rabbit SCs (XenoSC or alloSC, respectively) in a non-immunosuppressed rabbit PAD model.

## Methods

XMRCaps were produced using a modified alginate-poly-L-lysine-alginate microencapsulation method impregnating 12% v/v perfluorooctyl bromine (PFOB) and XenoSC or AlloSC. *In vitro *validations were performed in an agarose phantom consisting of four layers of 50, 100, and 200 XMRCaps. C-arm CT images (dynaCT, Siemens Artis Zee) were acquired and reconstructed at 0.46 mm isotropic voxel size. ^19^F 3T MRI was acquired with a 4-channel Tx/Rx ^19^F coil using a 3D TrueFISP sequence (Siemens Tim Trio, 4.1 ms TR; 2.0 ms TE; 70*° *FA; 1.3 mm isotropic voxel size; 32 averages). Reference ^1^H MRI was acquired with the system body or body matrix coil using a 3D gradient echo sequence. *In vivo *c-arm CT and MRI studies were performed at same day, one and two weeks after an intramuscular injection of 3 ml of XMRCaps in the hindlimb (n = 10) using identical imaging parameters as the in vitro studies (voxel size 1.5 × 1.5 × 2 mm). To test the repeatability of ^19^F MRI, the imaging sets were acquired twice on the same day in one rabbit with the coil repositioned in between. Reference markers with known PFOB concentrations were placed within the imaging field at the same depth of the injections relative to the coil to enable field inhomogeneity correction. Segmentation of the injection sites in the c-arm CTs and ^19^F MRIs was performed with the Otsu thresholding algorithm. Concentration was then determined by averaging the integrated ^19^F signal intensity over the segmented volume after normalization to standards.

## Results

**Figure 1:**
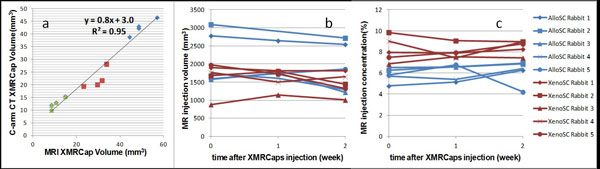
**(a) Correlation of the 200 (blue), 100 (red) and 50 (green) XMRCaps volumes in MRI vs. CT**. (b) The XMRCaps injection volume in 19MRI images at the day, one and two weeks after delivery. (c) Relative normalized fluorine concentration corresponding to the segmented volumes.

CT and MRI XMRCap volumes were highly concordant *in vitro *(y = 0.8x+3.0, R2 = 0.95) (Figure 1a). ^19^F MRI repeatability studies showed that the volume and concentration measurement errors were <3% and <6%, respectively. For the AlloSC rabbits, *in vivo *XMRCap injection volume and concentration decreased 0.2 ± 24% and 7.4 ± 17% respectively each week, compared to the XenoSC rabbits volume increased 1.2 ± 11% and concentration decreased 6.6 ± 17% each week (Figure 1b and 1c).

## Conclusions

MRI provides accurate assessment of XMRCap volumes, which were slightly larger than CT due to partial volume effects with the larger MRI voxel size. *In vivo *XMRCaps injection site volumes could be assessed on MRI and CT. However, only MRI was able to quantify the XMRCaps ^19^F concentration. Result shows that XMRCaps serial alternations with XenoSC and AlloSC are not significantly different, which demonstrates that XMRCaps prevent the immunorejection of the mismatched SCs.

## Funding

Siemens AG, NIH R33-HL089029, and the Maryland Stem Cell Research Foundation (2008-MDSCRFII-0399/2011-MDSCRFII-0043).

